# Fatal nicotine poisoning with an extremely high first available serum nicotine concentration and serial nicotine/cotinine measurements: A case report

**DOI:** 10.1016/j.toxrep.2026.102231

**Published:** 2026-02-24

**Authors:** Yuka Miyazaki, Jun Monma-Otaki, Sanae Kanno, Takuya Ito, Kenji Iwai, Ryohei Matsui, Tomonori Hattori

**Affiliations:** a Department of Advanced Emergency and Disaster Medicine, Nagoya City University Graduate School of Medical Sciences, Nagoya, Aichi, Japan; b Department of Forensic Medicine, Nagoya City University Graduate School of Medical Sciences, Nagoya, Aichi, Japan; c Department of Emergency and Critical Care Medicine, Nagoya City University Hospital, Nagoya, Aichi, Japan

**Keywords:** Nicotine poisoning, Toxicokinetics, Brain death, E-cigarette liquid, Gastric mucosal erosions

## Abstract

Human toxicokinetic data after massive nicotine exposure are scarce, and the relationship between circulating nicotine kinetics and organ-specific pathophysiology remains poorly understood, particularly in the post–cardiac arrest setting. We report a fatal case of a 36-year-old woman found collapsed with an empty 100-mL nicotine e-liquid bottle labeled 100 mg/mL (propylene glycol solvent) at the scene; the ingested volume and emesis were unknown. She was in asystolic cardiac arrest on emergency medical services arrival and achieved sustained return of spontaneous circulation after prolonged resuscitation. The first available serum nicotine concentration obtained on emergency department presentation was extremely high (6569.1 ng/mL). Serial measurements showed a marked decline in nicotine with rising cotinine, consistent with substantial metabolic conversion; however, toxicokinetic interpretation is constrained by sparse sampling under post–cardiac arrest physiology and potential hemodilution secondary to resuscitation. Early non-contrast head CT demonstrated diffuse cerebral edema, consistent with severe global hypoxic–ischemic injury; no nicotine-specific attribution for neurological outcome can be made in the absence of supportive biomarkers, electrophysiology, or neuropathology and given uncertainty in exposure timing. Endoscopy demonstrated acute gastric erosions without evidence of corrosive injury and with rapid healing. This case provides a detailed clinical and toxicological record of suspected massive nicotine exposure in a complex post–cardiac arrest setting, including serial nicotine/cotinine profiles and early organ-specific findings, with subsequent organ donation.

## Highlights

• First available serum nicotine concentration was 6569.1 ng/mL.
• Serial measurements showed nicotine decline with rising cotinine.
• Neurological injury was most consistent with severe post–cardiac arrest hypoxic–ischemic brain injury.
• Endoscopy revealed acute gastric erosions with rapid healing.
• The case illustrates major evidentiary limitations at extreme nicotine concentrations (semi-quantitative interpretation).

## Graphical Abstract

**Figure:**
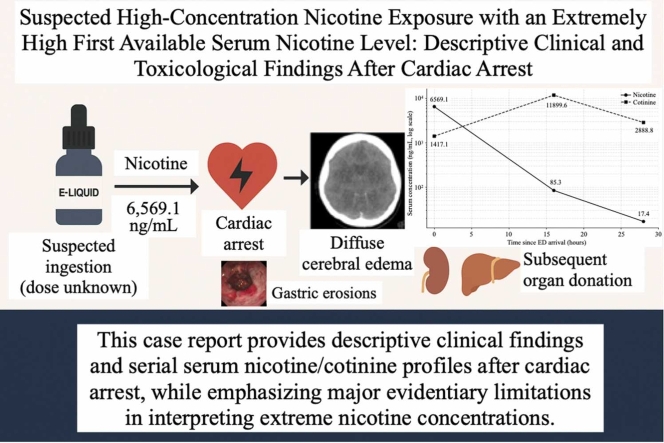

## 1 Introduction

The widespread availability of highly concentrated nicotine e-liquids, particularly through online marketplaces, has emerged as a significant source of severe, often fatal, poisoning [1]. Nicotine exerts potent agonistic and desensitizing effects on nicotinic acetylcholine receptors; massive exposure can precipitate cardiovascular collapse and profound neurological injury [2].

Surveillance data have shown an increase in reported exposures to e-cigarette liquids over the past decade, yet detailed human data on toxicokinetics (TK) and organ-specific toxicity after massive oral exposure remain limited [1], [3]. In particular, the relationship between circulating nicotine kinetics and downstream clinical outcomes is incompletely understood—especially in critically ill patients after cardiac arrest, where shock, vasopressor use, altered tissue perfusion, and fluid resuscitation may confound interpretation.

Here, we report a fatal case of suspected massive nicotine ingestion with an extremely high first available serum nicotine concentration and serial nicotine/cotinine measurements documenting nicotine decline with a concomitant rise in cotinine [3]. Despite improvement in several non-neurological organ dysfunctions, the patient developed irreversible neurological injury. Furthermore, we describe endoscopic gastric findings and document subsequent organ donation; however, we emphasize key evidentiary limitations and avoid transplantation outcome interpretation [4], [5].

### 1.1 Case presentation

A 36-year-old woman with a history of bipolar disorder was found outdoors at 02:17 in a state of collapse and coughing. Emergency medical services (EMS) were immediately activated by a bystander. The patient reportedly stated that she had “drunk something” and pointed to an empty 100-mL bottle labeled as nicotine e-liquid (100 mg/mL nicotine; propylene glycol solvent). Consequently, the exact volume of ingestion remained undetermined. Whether emesis occurred prior to EMS arrival remains unknown. On EMS arrival at 02:27, the patient was in asystolic cardiac arrest. She was transported with ongoing cardiopulmonary resuscitation and arrived at the emergency department (ED) at 02:44 with fixed dilated pupils. Following ED arrival, intermittent ROSC was achieved at 02:56; however, the clinical course was complicated by recurrent pulseless electrical activity (PEA) until sustained ROSC was finally established at 03:20. A non-contrast head CT was obtained at 03:30 (10 min after sustained ROSC) and showed diffuse cerebral edema.

On admission, she had severe mixed acidosis (pH 6.89) and required high-dose norepinephrine support for approximately 10 h after sustained ROSC. A qualitative urine drug screen was positive only for benzodiazepines. She had prescriptions for multiple psychotropic medications; however, actual adherence and recent co-ingestion could not be confirmed.

## 2 Toxicological analysis (LC–MS/MS)

Serum nicotine and cotinine concentrations were measured by liquid chromatography–tandem mass spectrometry (LC–MS/MS) (LCMS-8050, Shimadzu, Kyoto, Japan) following extraction via the QuEChERS (quick, easy, cheap, effective, rugged, and safe) method. Chromatographic separation was performed on a Kinetex XB-C18 column (2.1 mm i.d. × 100 mm, 2.6 μm; Phenomenex, Torrance, CA). The mobile phase consisted of (A) 10 mM ammonium formate with 0.1% formic acid and (B) methanol containing 10 mM ammonium formate with 0.1% formic acid. Gradient elution was used (B, 5%–95%) at a flow rate of 0.3 mL/min. The column temperature was maintained at 40°C, and the injection volume was 1 μL.

Mass spectrometric detection was performed using electrospray ionization in positive mode. Multiple reaction monitoring transitions were *m/z* 163.20→130.15 for nicotine, *m/z* 177.15→80.15 for cotinine, and *m/z* 290.15→154.05 for diazepam-d5 (internal standard). Calibration curves were linear over the calibration range up to 1 μg/mL for nicotine and cotinine (coefficient of determination, r² = 0.98 and 1.00, respectively). Samples with concentrations above the calibration range were diluted 10- or 20-fold with drug-free goat serum (Thermo Fisher Scientific, Waltham, MA) and re-analyzed. Formal assessments of analyte stability/volatility and extraction recovery were not performed.

## 3 Organ-specific findings and outcome

**Fig. 1:**
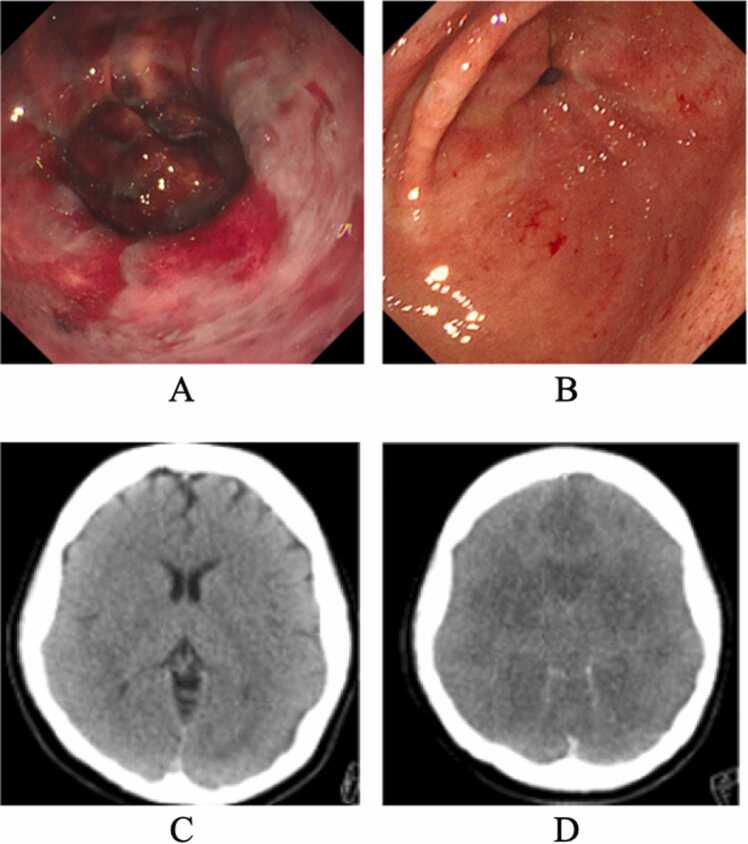
Clinical imaging findings. (A) Upper gastrointestinal endoscopy performed around 04:30 on day 0 (approximately 2 h after ED arrival and ∼70 min after sustained ROSC), revealing extensive hemorrhagic erosions in the gastric antrum. (B) Follow-up endoscopy on day 3 showing marked mucosal healing. (C) Non-contrast head CT obtained at 03:30 on day 0 (10 min after sustained ROSC) demonstrating diffuse cerebral edema. (D) Follow-up head CT on day 8 showing progression to brain herniation.

Upper gastrointestinal endoscopy performed around 04:30 on day 0 demonstrated extensive hemorrhagic erosions of the gastric antrum without evidence of corrosive injury (Fig. 1A), with marked healing on repeat endoscopy by day 3 (Fig. 1B). Transient hepatic and renal dysfunction improved during the clinical course. The patient remained in deep coma, and cerebral edema progressed to herniation by day 8 (Fig. 1D). The treating physicians confirmed clinical brain death based on absence of brainstem reflexes and a persistently flat electroencephalogram. After discussion with the family regarding organ donation, legal brain death determination was conducted in accordance with Japanese regulations. Following two mandatory assessments, multiple organs (including liver, kidney, heart, and lung) were recovered for transplantation after legal determination of brain death. However, tissue nicotine/metabolite concentrations were not assessed, and no inference can be made regarding tissue burden.

## 4 Discussion

This case provides descriptive observations in suspected massive nicotine exposure, including an extremely high first available serum nicotine concentration, serial nicotine/cotinine measurements obtained under post–cardiac arrest conditions, early neuroimaging after sustained ROSC, and endoscopic gastric findings. Because exposure timing and dose were unverified and the patient’s physiology was profoundly altered after cardiac arrest and shock, we present these findings as case-specific observations rather than generalizable clinical implications.

### 4.1 Serial nicotine/cotinine measurements in the post–cardiac arrest setting

**Fig. 2:**
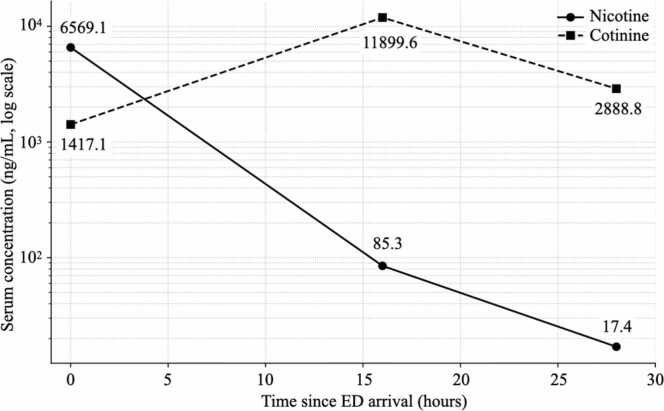
Serum nicotine and cotinine concentration–time profiles. Serum nicotine concentrations exhibited a marked decline (6569.1 ng/mL at 0 h to 85.3 ng/mL at 16 h and 17.4 ng/mL at 28 h), whereas cotinine levels peaked at 16 h (1417.1 ng/mL at 0 h to 11,899.6 ng/mL at 16 h) before subsequently decreasing (2888.8 ng/mL at 28 h). Time 0 denotes the first sample obtained on ED arrival; subsequent samples were collected on the next morning and the following morning (approximately 16 and 28 h). Concentrations were measured in serum by LC–MS/MS (LCMS-8050, Shimadzu) after QuEChERS extraction using diazepam-d5 as an internal standard; samples above the calibration range (up to 1 μg/mL) were diluted 10- or 20-fold with drug-free goat serum and re-analyzed. Reported concentrations were not adjusted for potential hemodilution secondary to resuscitative efforts and fluid administration.

The most notable toxicological finding was the extremely high first available serum nicotine concentration (6569.1 ng/mL). However, it must be acknowledged that formal validation of analyte stability and extraction recovery was not performed at this extreme concentration; thus, this value should be interpreted as a semi-quantitative indicator of the massive scale of exposure rather than a precise absolute concentration. This finding was followed by a marked decline in nicotine with a concomitant rise in cotinine (Fig. 2), consistent with substantial metabolic conversion via hepatic pathways such as CYP2A6 [3]. However, interpretation of TK parameters should remain cautious. Sampling points were limited, and the patient was critically ill after cardiac arrest with shock and vasopressor support—conditions that can alter distribution, tissue perfusion, and apparent clearance. In addition, concentrations were not corrected for potential hemodilution related to resuscitation. Consequently, we present the observed kinetic pattern (nicotine decline with concomitant cotinine rise) as a descriptive observation rather than asserting a precise elimination half-life or providing definitive evidence regarding the saturation of clearance pathways.

### 4.2 Neurological injury: findings consistent with severe hypoxic-ischemic brain injury

Early diffuse cerebral edema was present on head CT shortly after sustained ROSC (Fig. 1C). The patient experienced asystolic cardiac arrest with profound acidemia (pH 6.89), repeated ROSC/PEA episodes, and vasopressor-dependent shock, all of which can produce early cerebral edema through global ischemia–reperfusion injury and post–cardiac arrest pathophysiology. Accordingly, the neuroimaging findings and subsequent progression to brain death are most consistent with severe hypoxic–ischemic brain injury. No nicotine-specific attribution for neurological outcome can be made in this report because exposure timing was unverified and supportive biomarkers (e.g., NSE, S100B), electrophysiology, or neuropathology were not available [6], [7]. The patient was found at 02:17 and was in asystolic cardiac arrest when EMS arrived at 02:27; however, the timing and amount of ingestion and the presence of emesis were unknown. Therefore, the interval from ingestion to cardiac arrest and the specific sequence of pathophysiological events remain undetermined, and our observations are limited to the post-arrest clinical course. We include these early neuroimaging findings to document the neurological trajectory in suspected massive nicotine exposure after cardiac arrest, while emphasizing that etiologic attribution beyond hypoxic–ischemic injury is not supported in this case.

### 4.3 Gastrointestinal and solid-organ findings: severe but clinically reversible injury

Upper gastrointestinal endoscopy performed around 04:30 on day 0 demonstrated extensive hemorrhagic erosions in the gastric antrum with marked healing by day 3 (Fig. 1A–B). The endoscopic appearance and rapid improvement were consistent with acute mucosal injury without overt corrosive burn. Potential mechanisms include mucosal ischemia and stress-related injury in the setting of shock and catecholamine exposure; a direct local effect of nicotine or solvent constituents cannot be excluded [8].

Transient hepatic and renal dysfunction improved during the clinical course. Multiple organs were recovered for transplantation after legal determination of brain death.

## 5 Limitations

Exposure ascertainment was limited: the bottle was empty, but the ingested volume and emesis were unknown, and co-ingestion could not be fully excluded despite urine screening being positive only for benzodiazepines and medication adherence being uncertain. Toxicokinetic interpretation is constrained by sparse sampling and post–cardiac arrest physiology, vasopressor use, and potential hemodilution. Direct nicotine-specific neurotoxicity cannot be established in this report without supportive biomarkers, electrophysiology, or neuropathology; therefore, etiologic attribution beyond post–cardiac arrest hypoxic–ischemic injury is not supported in this case. Furthermore, as a single case report, generalizability is limited and threshold-based conclusions should be avoided.

Finally, assay performance characteristics such as analyte stability/volatility and extraction recovery were not independently evaluated, which may limit strict quantitative interpretation at very high concentrations.

## 6 Conclusion

This case report describes suspected massive nicotine ingestion with an extremely high first available serum nicotine concentration and serial nicotine/cotinine measurements showing nicotine decline with rising cotinine under post–cardiac arrest conditions. Neurological injury progressed to brain death and was most consistent with severe hypoxic–ischemic brain injury. Endoscopy revealed acute gastric mucosal erosions with rapid healing, and transient hepatic and renal dysfunction improved during the clinical course. In conclusion, we report the clinical and toxicological findings of a rare case of suspected fatal nicotine exposure with an extremely high first available serum nicotine concentration. These descriptive observations provide a detailed record of the clinical trajectory and serial nicotine/cotinine profiles in a complex post–cardiac arrest setting.

## 7 Declarations

### 7.1 Patient consent

Written informed consent for publication of clinical details and images was obtained from the patient’s legal next of kin (mother) using an institutional case-report consent form at Nagoya City University Hospital (dated July 7, 2024). The signed form authorizes publication in peer-reviewed medical journals and is held by the authors.

## CRediT authorship contribution statement

**Tomonori Hattori:** Writing – review & editing, Visualization, Supervision, Conceptualization. **Sanae Kanno:** Validation, Formal analysis. **Jun Monma-Otaki:** Validation, Formal analysis, Data curation. **Yuka Miyazaki:** Writing – original draft, Data curation, Conceptualization. **Ryohei Matsui:** Writing – review & editing. **Kenji Iwai:** Writing – review & editing. **Takuya Ito:** Writing – review & editing.

## Ethical approval and consent for publication

Ethical approval was not required for this single case report.

## Declaration of Generative AI and AI-assisted technologies in the writing process

During the preparation of this manuscript, the authors used the AI-based tool ChatGPT (OpenAI) to assist with language editing, figure formatting, and structural refinement. The authors reviewed and edited the content generated by the tool and take full responsibility for the final manuscript.

## Funding

This research did not receive any specific grant from funding agencies in the public, commercial, or not-for-profit sectors.

## Declaration of Competing Interest

The authors declare that they have no known competing financial interests or personal relationships that could have appeared to influence the work reported in this paper.

## Data Availability

Data will be made available on request.
